# Peptide Neurotoxins that Affect Voltage-Gated Calcium Channels: A Close-Up on ω-Agatoxins

**DOI:** 10.3390/toxins3010017

**Published:** 2011-01-04

**Authors:** Emilie Pringos, Michel Vignes, Jean Martinez, Valerie Rolland

**Affiliations:** IBMM, UMR 5247, CNRS, Max Mousseron Institut of Biomolecules, Montpellier 1 &amp; 2, Place E. Bataillon, Montpellier, France; Email: epringos@univ-montp2.fr (E.P.); michel.vignes@univ-montp2.fr (M.V.); jean.martinez@univ-montp1.fr (J.M.)

**Keywords:** ω-agatoxin IV B, peptide neurotoxins, voltage-gated calcium channels

## Abstract

Peptide neurotoxins found in animal venoms have gained great interest in the field of neurotransmission. As they are high affinity ligands for calcium, potassium and sodium channels, they have become useful tools for studying channel structure and activity. Peptide neurotoxins represent the clinical potential of ion-channel modulators across several therapeutic fields, especially in developing new strategies for treatment of ion channel-related diseases. The aim of this review is to overview the latest updates in the domain of peptide neurotoxins that affect voltage-gated calcium channels, with a special focus on ω-agatoxins.

## 1. Introduction

Numerous peptides found in animal venom have become invaluable tools for the study of the physiological role and structure of the channels and receptors. Peptide toxins with nanomolar affinities for corresponding targets proved to be useful agents for discriminating between different types of native ion channel currents. Regarding the relatively small size of peptide toxins, these can be obtained by chemical synthesis or recombinant methods, which represent an advantage in comparison to extraction from large quantities of venom which is often hardly accessible. Moreover, their target specificity and relatively high biological stability makes them a very rewarding class of molecular probes for use in the investigation of channels and receptors. 

Understanding the importance and impact of ion channels as therapeutic targets may lead to the discovery of a new class of medications. This explains the particular interest in clinical application of toxins; some of them or their synthetic analogues are in clinical phases of trial. Others are already on the market, protected by international patents, where their properties are used to treat numerous channel-related diseases, such as: neurological disorders, cancer and cardiovascular system anomalies [1,2]. 

Even though only a very small percentage of venom compounds are characterized pharmacologically, the range of toxins affecting various types of channels and receptors is immensely broad. The voltage-sensor trapping mechanism may be a common mode of action for toxins acting on all voltage-gated ion channels. Much effort in this field is dedicated to the pharmacology of toxins that modify channel’s properties. The main usefulness of peptide toxins concerns their high specificity towards the corresponding voltage-gated calcium channels; this feature allows a distinction to be drawn from calcium currents, and is, in some cases, the only available pharmacological criterion of difference. 

## 2. Voltage-Gated Ion Channels: NaV, CaV, KV

**Figure 1:**
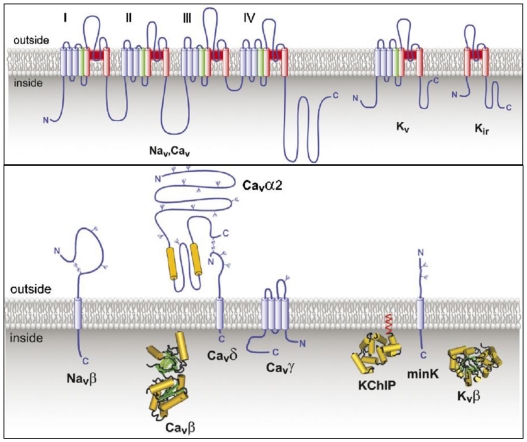
The topological structure of voltage-gated ion channels. a. (top) The structurally similar voltage-gated ion channels Na_V_, Ca_V_ and K_V_. Pore-forming transmembrane alpha helices S_5_-S_6 _are represented as red cylinders, voltage sensor segment S_4 _as green and the rest of the transmembrane segments S_1_-S_3_ as blue. b. (bottom) All transmembrane (Na_V_β, Ca_V_α_2_, Ca_V_δ, Ca_V_γ, minK) and predicted three-dimensional intracellular (Ca_V_β, KChIP, K_V_β) auxiliary subunits of the voltage-gated ion channels Na_V_, Ca_V_, and K_V._ Cylinders illustrate predicted alpha helices of the subunits, small blue “ψ” represent N-linked carbohydrate chains [3].

Voltage-gated ion channels regulate fast, potential-dependent changes in ion permeability in cells. Besides being involved in nerve message transmission, they initiate a number of important intracellular mechanisms such as muscle contraction, neurotransmitter and hormone release. We can distinguish three types of voltage-dependent cation channels: sodium, potassium and calcium. All of these channels belong to the family of tetrameric cation channels and share structural similarities: they contain four membrane-spanning subunits or domains (I-IV) surrounding a central pore that is selective for cations of one kind or another. They also possess various auxiliary subunits [3]. A representation of the structure of voltage-gated ion channels is presented in Figure 1.

The structurally similar voltage-gated ion channels Na_V_, Ca_V_ and K_V_ are illustrated in Figure 1(a). The pore-forming transmembrane alpha helices S_5_-S_6 _are represented as red cylinders, the voltage sensor segment S_4 _as green and the rest of the transmembrane segments S_1_-S_3_ as blue. All transmembrane (Na_V_β, Ca_V_α_2_, Ca_V_δ, Ca_V_γ, minK) and predicted three-dimensional intracellular (Ca_V_β, KChIP, K_V_β) auxiliary subunits of the voltage-gated ion channels Na_V_, Ca_V_, and K_V_ are depicted in Figure 1(b). The cylinders illustrate the predicted alpha helices of the subunits, while the small blue “ψ” represent the N-linked carbohydrate chains [3]. 

## 3. Voltage-Gated Calcium Channels: CaV

Voltage-gated calcium channels play an important role in neuronal communication and neurotransmitter release. Thus, they are molecular targets for pharmacological agents as well as for a broad range of potent neurotoxins [3]. Synaptic transmission is not the only process regulated by modulation of calcium concentration; changes in the distribution of calcium ions initiate many other biological processes in living organism such as muscles contraction, gene expression, secretion and serve as regulator of calcium-dependent second messenger cascades [4]. 

**Figure 2:**
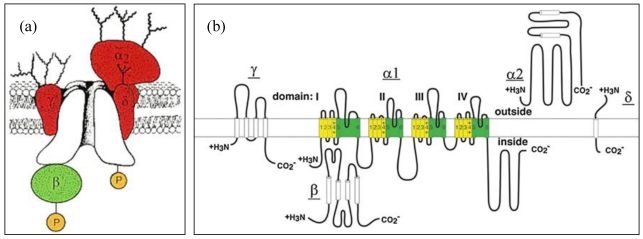
The topological structure of voltage-gated calcium channels(Ca_V_) [5].

Although physiological and pharmacological properties of voltage-gated calcium channels can be very different, they share a common ensemble of structural motives. Various types of calcium channels have the same general structure; they are heteromeric proteins composed of a pore-forming subunit α_1_, a transmembrane δ subunit that is disulfide-linked to an α_2_ protein, an intracellular β subunit, and another transmembrane γ subunit. All these subunits form a fully functional calcium channel, as illustrated in Figure 2 [5]. The α_1 _subunit is the largest protein that composes the channels; it contains the conduction pore, the gating mechanism and the voltage sensor. In addition, some of the sites of channel regulation (by second messengers, toxins or drugs) are also located on the α_1_ subunit. Amino acid sequencing predicts that the α_1_ subunit forms a transmembrane protein, which is organized in four repeated domains. Each domain comprises six segments of α helical structure, as illustrated in Figure 2(b) with green cylinders for the pore-forming segments and yellow cylinders for the sensor segments and a loop [5]. Both N- and C-termini of the protein are located on the intracellular side of the membrane. 

The α_1_ subunit is expressed in living cells without other subunits and forms fully functional calcium channels, however, with the kinetics and voltage dependency of currents, which differs from native channels. Up to date, we can distinguish 10 different isoforms of α_1_ subunit, which can be divided into three functionally and structurally related families: Ca_V_1, Ca_V_2 and Ca_V_3 [6]. The similarity of amino acid sequence within a family comes to more than 70%, but among the families this similarity drops to less than 40% [7].

**Table 1:** Different classification and nomenclature of calcium channels [8].

Superfamily	Family	Former Name	Proposed Name	Pharmacology
HVA	L	α_1C_	Ca_v_1.2	dihydropyridines, phenyl-alkylamines, benzodiazepinesand other small molecules
		α_1D_	Ca_v_1.3	
		α_1F_	Ca_v_1.4	
		α_1S_	Ca_v_1.1	
	N	α_1B_	Ca_v_2.2	ω−conotoxin GVIA
	P/Q	α_1A_	Ca_v_2.1	ω-agatoxin IVA
	R	α_1E_	Ca_v_2.3	SNX-482
LVA	T	α_1G_	Ca_v_3.1	No selective blockers reported to date
		α_1H_	Ca_v_3.2	
		α_1I_	Ca_v_3.3	

Six different types of voltage-dependent calcium channels have been identified: L-, N-, P-, Q-, R- and T-type (Table 1) [8]. The L-, N-, P-, Q- and R-type calcium channels are activated at high voltage (HVA) while T-type calcium channel is activated at negative membrane potentials and is the only member of the low voltage-activated (LVA) family. All members of the HVA family share some common features, such as requiring strong depolarization for their activation, but their pharmacological properties are completely different. Several studies have revealed that L-type channels are sensitive to a certain number of small organic molecules and to calciseptine, a neurotoxin from the venom of black mamba [9]. N-type calcium channels are the main target of ω-conotoxins, while P/Q-type channels are selectively blocked by ω-agatoxins. No antagonists were known for R-type calcium channels until the discovery of SNX-482, a peptide neurotoxin found in the venom of a tarantula [10]. Moreover, multiple isoforms of R-type calcium channels α_1Ε_ subunit have been described, with different pore properties [11].

**Figure 3:**
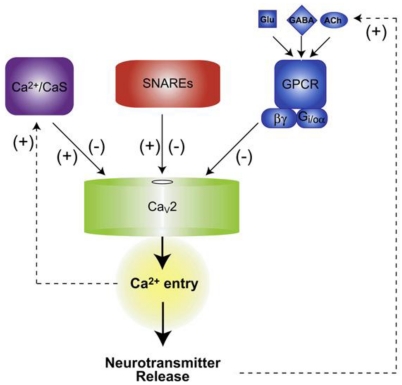
A regulatory network that controls neurotransmitter release: The pathway diagram of the Ca^2+^ channel regulatory network [5].

As shown in Figure 3, Ca^2+^ entry into the presynaptic terminal and neurotransmitter release are regulated by three signaling pathways. Firstly, activation of calmodulin (CaM), calmodulin-like nCaS protein and Ca^2+^/CaM-dependent protein kinase II (CaMKII) directly, or indirectly by the presence of Ca^2+^ ions in their environment, lead to enhancement or inhibition of voltage-gated calcium channels (purple). Secondly, SNARE proteins, both by binding as individual target SNAREs (syntaxin or SNAP-25) or as a complete SNARE complex, are able to activate or inhibit voltage-gated calcium channels and, thus, control the entry of Ca^2+^ ions into the presynaptic terminal (red). Finally, neurotransmitters like glutamate, GABA, and acetylcholine activate βγ subunits of the G_i/o_ protein (blue). Then these subunits inhibit voltage-gated calcium channels and, thus, the entry of Ca^2+^ ions into the presynaptic terminal [5].

## 4. Peptide Modulators of Voltage-Gated Calcium Channels

Diverse animal species evolved a broad range of toxic compounds, which are used in their natural environment for defense and pray immobilization. Venoms of some predatory animals are extremely toxic; they paralyze hunted preys within seconds and, in some cases, may be very dangerous, even for humans. 

Animals such as snakes, scorpions, spiders and cone snails arouse researchers’ interest, because their venoms have proved to be a rich source of highly specific ligands for a variety of pharmacological targets. Most crude venoms contain a complicated mixture of biologically active substances, like proteins, peptides, polyamines, enzymes, nucleic acids and various small organic molecules. Recent developments of analytical and synthetic methods have permitted the isolation, identification and synthesis of many novel peptide toxins, which revealed properties of voltage-gated calcium channel blockers (Table 2). 

**Table 2:** Commonly used peptide toxins and their specificity.

Channel Type	Channel Blocker	Effective Concentration Allowing Selectivity
P-type	ω-Agatoxin IVB	IC_50_ = 15 nM
	ω-Agatoxin IVA	K_d_ = 2 nM
N-type	ω-Conotoxin GVIA	IC_50_ = 100 nM-2 µM
	ω-Conotoxin MVIIA	IC_50_ = 100 nM-1 µM
	ω-Conotoxin SVIB	IC_50_ = 100 nM-2 µM
L-type	Calciseptine	IC_50_ = 15 nM
	Calcicludine	IC_50_ = 0.2 nM
Q-type	ω-Conotoxin MVIIC	IC_50_ = 50 nM-1 µM
	ω-Agatoxin IVA	K_d_ = 100-200 nM
R-type	SNX-482	IC_50_ = 15-30 nM

### 4.1. Peptide Toxins from Scorpion Venom

Numerous peptide neurotoxins affecting voltage-gated channels have been isolated from scorpion venoms. One of these, kurtoxin is a 63 amino acid peptide (Table 3), isolated from *Parabuthus transvaalicus* presenting a sequence more closely resembling the scorpion α-toxins that target sodium channels rather than the known calcium channel gating modifier toxins [12]. 

**Table 3:** Kurtoxin peptidic sequence.

	Sequence	Swiss-Prot
Kurtoxin	KIDGYPVDYWN**C**KRI**C**WYNNKY**C**NDL**C**KGLKADSGY**C**WGWTLSCY**C**QGLPDNARIKRSGR**C**RA	P58910

At first, kurtoxin was defined as a specific blocker of T-type voltage-gated calcium channels, but recent studies have shown that kurtoxin also affects L-, N-, and P/Q-type voltage-gated calcium channels in neurons [13]. Thus, kurtoxin is no longer considered specific and can only be used in R-type voltage-gated calcium channels studies.

### 4.2. Peptide Toxins from Snake Venom

An abundance of peptide toxins have been identified in snake venoms. Among these, calciseptine, calcicludine and various cysteine-rich secretory proteins (CRISPs) are mainly L-type calcium channel modulators. 

#### 4.2.1. Calciseptine

Calciseptine is a natural peptide consisting of 60 amino acids (Table 4) with eight cysteines forming four disulfide bonds, isolated from the venom of the black mamba (*Dendroaspis polylepis*). The peptide is a natural L-type calcium channel blocker in heart and smooth muscle cells. Recently, it was shown that calciseptine also acts as a calcium channel agonist in skeletal muscles, increasing calcium currents by around 40% [14], but it clearly does not affect N- nor T-type calcium channels. Observations *in vitro* and *in vivo* suggest that calciseptine shares the properties of 1,4-dihydropyridine derivatives in modulating the permeation of divalent cations through L-type calcium channels. It seems that the channel sensitivity to calciseptine is tissue-dependent and higher in cardiovascular system cells, showing an IC_50_ of 15 nM [9].

#### 4.2.2. Calcicludine

Calcicludine is a 60 amino acid peptide (Table 4), with six cysteines forming three disulfide bonds, isolated from the venom of the green mamba (*Dendroaspis angusticeps*). Calcicludine potentially blocks all types of HVA calcium channels (L-, N- and P-type) in a variety of excitable cells. Voltage-clamp experiments have shown high affinity blocking in the 0.2 nM range with a tissue- and species-dependent sensitivity [15]. L-type calcium channels in cerebellar granular cells and cardiac myocytes seem to be the main target of calcicludine. As shown, calcicludine triggers a rapid, irreversible decrease of L-type calcium channel peak current amplitude but even upon saturating doses the blocking is incomplete, with a maximum inhibition of 58% [16].

**Table 4:** Calciseptine and calcicludine peptidic sequences.

	Sequences	Swiss-Prot
Calciseptine	RI**C**YIHKASLPRATKT**C**VENT**C**YKMFIRTQREYISERG**C**G**C**PTAMWPYQTE**CC**KGDR**C**NK	P22947
Calcicludine	WQPPWY**C**KEPVRIGS**C**KKQFSSFYFKWTAKK**C**LPFLFSG**C**GGNANRFQTIGE**C**RKK**C**LGK	P81658

### 4.3. Peptide Toxins from Marine Worm

Glycerotoxin (GLTx) is a 320 kDa protein extracted from the venom of the marine blood worm *Glycera convulata* [17]. This neurotoxin triggers a potent enhancement of calcium currents through N-type voltage-gated calcium channels and appears to elicit (among others) glutamate release in rat brain synaptosomes [18].

### 4.4. Peptide Toxins from Conus Snails

Cone snails of the genus *Conus* are a diverse group of predatory gastropod mollusks that hunt using a venomous cocktail comprising more than a hundred peptides. Peptides from *Conus* venoms are generally small, 10-30 amino acids, and rich in disulfide bonds, often containing unusual post-translationally modified amino acids. A unique feature of conotoxins is their specific action on a number of different ion channels and receptors, thus, they are widely used in neuroscience research. ω-Conotoxins were the first natural substances discovered that selectively affect neuronal voltage-sensitive calcium channels found in mammalian cells [19]. Further investigations concerning the composition of *Conus* venom allowed the identification of several other classes of polypeptides. These were classified according to their affinity towards different targets.

Originating from different species of marine snails, ω-conotoxins exhibit interspecies sequence variation and show less than 30% identity [20]. Not taking into consideration the cysteine residues at positions 1, 8, 15, 16, 20 and 27, the only conserved amino acid, among all ω-conotoxins, is a glycine at position 5 (Table 5). However, comparison of N-terminus part reveals a degree of sequence conservation expected between homologous proteins descending from the same genus. Although a lack of general resemblance, ω-conotoxins share a number of similarities. All of them are peptides with a relatively high number of basic amino acids, which results in a net positive charge of the toxin ranging from +5 to +7. Moreover, the overall positive net charge of the toxin is enhanced by post-translational amidation of the C-terminus [21].

**Table 5:** ω−Conotoxins CVID, GVIA, MVIIA, MVIIC and SVIB.

ω-Conotoxins	Sequences	Swiss-Prot
CVID	**C**KSKGAK**C**SKLMYD**CC**SGS**C**SGTVGR**C**	P58920
GVIA	**C**KS PGSS**C**SPTSYN**CC**RS**C**NPYTKR**C**Y	P01522
MVIIA	**C**KGKGAK**C**SRLMYD**CC**TGS**C**RSGK**C**	P05484
MVIIC	**C**KGKGAP **C**RKTMYD**CC**SGS**C**GRRGK**C**	P37300
SVIB	**C**KLKGQS**C**RKT SYD**CC**SGS**C**GRSGK**C**	P28881

**Figure 4:**
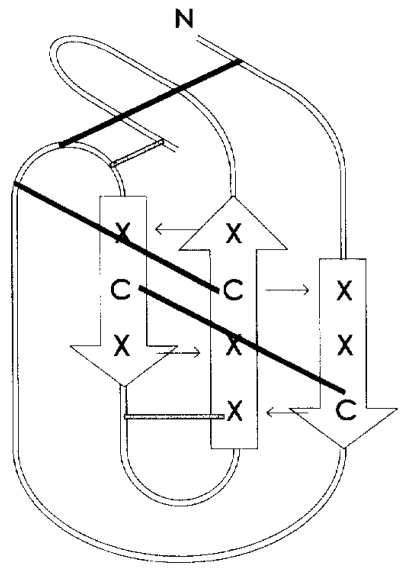
Schematic diagram of the “inhibitor cystine-knot” (ICK) motif illustrating the disulfide bridges as black bars, half-cystine residues as C and the other β-strand residues as X [26].

Based on a characteristic arrangement of cysteine residues, three structurally different classes of venom peptides can be defined with two, three and four internal loops. Striking structural motive is represented by characteristic patterns of arrangement of cysteine residues, which can be found not only in ω-conotoxins, but also in other peptide classes isolated from venoms of different species. These small peptides adopt a specific, relatively rigid conformation, which is based on the arrangement of three disulfide bridges that shape a structural framework. Cysteines arranged in a four-loop framework, the so-called cystine-knot motif (Figure 4), is common to a number of toxic and inhibitory peptides [22].

N-type calcium channels are specifically inhibited by one of the first components isolated from the venom of the marine snail *Conus geographus*, ω-conotoxin GVIA, which was extensively used to identify and characterize these channels in various cell types [19,23]. Expanding interest in this field of research led to the discovery of an additional ω-conotoxin MVIIA, originating from *Conus magus*. Despite the low sequence identity, both ω-conotoxins target N-type calcium channels [24]. ω-Conotoxin MVIIA was shown to be less selective than ω-conotoxin GVIA, but it dissociates quicker from the receptor and, thus, was a more promising drug candidate [2,25]. From ω-conotoxin MVIIA, the ziconotide (Prialt®) was synthesized and approved, in 2004, for the treatment of pain [2]. The more recently isolated ω-conotoxin CVID from the venom of *Conus catus* is even more selective toward the N-type voltage sensitive calcium channels than others ω-conotoxins characterized to date [26]. At the same time, ω-conotoxin CVID reveals a low potency for the P/Q-type calcium channels, and, thus, may be a big step towards a “second generation” of medical application of ω-conotoxins that will hopefully overcome some of the side effects presently associated with the clinical use of ω-conotoxin.

In contrast to ω-conotoxin GVIA, as shown in Table 5, ω-conotoxin MVIIA has high sequence identity with ω-conotoxin MVIIC, which, despite being of the same origin, possess quite different selectivity. ω-Conotoxin MVIIC (a 26 amino acid peptide) and the functionally related ω-conotoxin SVIB (a 26 amino acid peptide isolated from *Conus striatus*) reversibly block both N-type and P/Q-type calcium channels [27,28]. Additional types of toxins, which act non-specifically on the N-type calcium channels, as well as on the P/Q-type, are: ω-agatoxin IIIA and ω-grammotoxin SIA. Therefore, a given channel may have multiple binding sites for different toxins, or several different toxins may target the same binding site.

### 4.5. Peptide Toxins from Spider Venom

#### 4.5.1. Structure of the Spider Venom Peptides

The expression of spider genes provides precursors of the venom peptide toxins [29]. Precursors are composed of an N-terminal signal peptide, a highly variable length propeptide region rich in acidic residues and the mature toxin sequence. Precursors undergo post-translational modifications and release the mature venom peptide toxins. The mature peptide sequence has evolved through time and within toxin superfamilies, but the cystine framework remains strictly unchanged (Figure 4). 

Most ion channel peptide toxins, including those from spiders [30], have, in addition to modified N- and C-termini for increased *in vivo* stability, several disulfide bonds adopting a structural motif designated as “inhibitor cystine-knot” (ICK) motif, which confers a constrained globular conformation to the molecule. The common configuration of this motif consists of a “triple-stranded, anti-parallel β-sheet stabilized by a cystine knot” presenting the following amino acid consensus sequence: CX_3-7_CX_3-6_CX_0-5_CX_1-4_CX_4-13_C, where X can be any amino acid [31]. Usually only three disulfide bridges constitute the cystine knot, but in some cases a fourth one exists that stabilizes the fifth loop, for example in ω-agatoxin IVB. 

Structurally, the ICK motif consists of an anti-parallel, triple stranded β-sheet stabilized by two disulfide bridges forming a ring through which the third disulfide bond passes. The length of the first two β-strands is usually three residues and the length of the hairpin last β-strand is four residues.

#### 4.5.2. Spider Peptide Toxins with Action on Voltage-Gated Calcium Channels

Several peptide toxins acting on voltage-sensitive calcium channels have been isolated from spider venoms [32]. These channels play a fundamental role in cardiac, muscular and neuronal functions. As reported in many studies, some peptide toxins are able to nonspecifically affect multiple types of voltage-gated ion channels, even from different families. These toxins obviously recognize a common conserved pattern present on the channels. Some toxins, such as ω-atracotoxins from *Hadronyche* species [33,34,35,36], toxins from *Hololena curta* [37,38] and Plectreurys toxin II (PLTX-II) [39], modulate insect voltage-gated calcium channels and are useful in research of novel insecticides, while other toxins have interesting, more or less specific, activities on mammalian voltage-gated calcium channels. *Hololena curta* venom, in addition to it insecticidal activity, is a potent inhibitor of voltage sensitive calcium channels and neurotransmitter release in mammalian brain synaptosomes [40]. PLTX-II is a 44 amino acid peptide toxin, isolated from the venom of the hunting spider *Plectreurys tristis*, which also acts on both insect and mammalian voltage-gated calcium channels [41].

##### 4.5.2.1. Phonetoxins

PhTx3 is a protein fraction isolated from the venom of the Brazilian aggressive “armed” spider *Phoneutria nigriventer* (Table 6). Sub-fractions of PhTx3 such as Tx3-3, Tx3-4 and Tx3-6 block voltage-gated calcium channels. Recent work has revealed the neuroprotective role of PhTx3 due to the potent calcium channel inhibition [42]. In GH3 cells, Tx3-1 was shown to increase the frequency of calcium oscillations, while Tx3-2 partially blocks L-type calcium channels.

**Table 6:** Sequences of phonetoxins PhTx3 and PRTx3-7 affecting voltage-gated calcium channels [49].

Phonetoxins	Sequences	Swiss-Prot
Tx3-1	AE**C**AAVYER**C**GKGYKR**CC**EERP**C**K**C**NIVMDN**C**T**C**KKFISE	O76200
Tx3-2	A**C**AGLYKK**C**GKGASP**CC**EDRP**C**K**C**DLAMGN**C**I**C**K	O76201
Tx3-3	G**C**ANAYKS**C**NGPHT**CC**WGYNGYKKA**C**I**C**SGXNWK	P81789
Tx3-4	S**C**INVGDF**C**DGKKDD**C**Q**CC**RDNAF**C**S**C**SVIFGYKTN**C**R	P81790
	CEVGTTATSYGI**C**MAKHK**C**GRQTT**C**TKP**C**LSKR**C**KKNH	
Tx3-6	A**C**IPRGEI**C**TDD**C**E**CC**G**C**DNQ**C**Y**C**PPGSSLGIFK**C**S**C**AHANKYF**C**NRKKEK**C**KKA	P81792
PRTx3-7	A**C**AGLYKK**C**GKGVNT**CC**ENRP**C**K**C**DLAMGN**C**I**C**KKKFVEFFGG	P83911

Early studies of Tx3-3 (also named ω-PnTx3-3) revealed that its effect on blocking glutamate and acetylcholine release is additive to that of ω-agatoxin IVA. In brain cortical synaptosomes, Tx3-3 blocks P/Q-type calcium channels with an IC_50_ of 0.9 nM [43]. More recent electrophysiology experiments show that Tx3-3 also blocks P/Q- and R-type currents in cerebellar granule cells with an IC_50_ of 12 nM, while other calcium channels were partially affected in other systems [44].

Another sub-fraction of PhTx3 called Tx3-4 or ω-Phonetoxin IIA also blocks P/Q-type calcium channels in brain synaptosomes with an IC_50_ of 7.9 nM [45]. Electrophysiology tests revealed the blocking of N- and P/Q-type calcium channels. Tx3-4 has a neuroprotective effect on hippocampal slices submitted to ischemia induced oxygen/glucose deprivation [46]. Furthermore, it appears that the toxin binds at more than one site and that the ω-conotoxins GVIA or MVIIC displace it only partially. Tx3-4 also decreases glutamate release by blocking the carriers in synaptosomes in a time-dependent, calcium-independent way [47]. Recent studies show that Tx3-4 inhibits calcium channels coupled to KCl-induced exocytosis of synaptic vesicles with an IC_50_ of 1.1 nM and that the P/Q-type calcium channels are the main target of the toxin [48]. 

As previously described for subfractions of PhTx3, Tx3-6 affects N-, P/Q- and R-type voltage-gated calcium channels but Tx3-6 toxin exhibited the highest potency against N-type (conotoxin GVIA sensitive) current [50]. Tx3-6, patented with the name of Phα1β, was more effective than ω-conotoxin MVIIA not only to prevent, but especially to reverse, previously installed persistent chemical and neuropathic pain, having a therapeutic index wider than w-conotoxin MVIIA [51]. Tx3-6 (also called PnTx3-6) shows 58% identity with ω-agatoxin IIIB [45]. Agatoxins are known to act on calcium channels and, indeed, Tx3-6 inhibits N-, P/Q- and R-type voltage activated calcium channels [50]. More acute research demonstrate that Tx3-6 blocks calcium-induced glutamate release involving P/Q-type calcium channels with an IC_50_ of 263 nM but does not affect glutamate synaptosomal secretion.

From the venom of another Brazilian spider, *Phoneutria reidyi,* a 4 kDa peptide was isolated and called PRTx3-7. According to a recent publication [52], the toxin seems to trigger an incomplete blocking of L-, R-, P/Q- and with best affinity N-type calcium channels, without affecting the channel kinetics. Currents partially recover after wash-out. 

##### 4.5.2.2. ω-Filistatoxin-Kh1a

Like PRTx3-7, ω-filistatoxin-Kh1a (also called ω-FLTX-Kh1a and DW13.3) is not a very selective toxin. It is a 74 amino acid peptide toxin with a sequence containing 12 cysteines isolated from the venom of *Filista hibernalis *(Table 7). As some early studies have revealed, ω-FLTX-Kh1a blocks all types of native calcium channels except from T-type [53].

**Table 7:** Filistatoxin peptidic sequence.

	Sequence	Swiss-Prot
ω-FLTX-Kh1a	AE**C**LMIGDTS**C**VPRLGRR**CC**YGAW**C**Y**C**DQQLS**C**RRVGRKRE**C**GWVEVN**C**K**C**GWSWSQRIDDWRADYS**C**K**C**PEDQ	P60979

##### 4.5.2.3. SNX-325

A 49 amino acid peptide has been isolated from the spider *Segestria florentina* venom. SNX-325 is an N-type calcium channel antagonist with the same cysteine pattern as ω-agatoxin IVA [54]. At micromolar concentrations, SNX-325 affects most calcium channels, including P/Q-type, but at nanomolar concentrations it selectively inhibits N-type calcium channels. 

##### 4.5.2.4. SNX-482

The peptide neurotoxin SNX-482 is a 41 residue acidic peptide containing three disulfide bonds isolated from the venom of the African tarantula *Hysterocrates gigas* [55]. SNX-482 is also the first known selective antagonist of R-type calcium channels. *In vitro* studies have shown that SNX-482 is a potent and selective inhibitor of R-type calcium channels. It appears that it blocks some native R-type currents, depending on which α_1E_ subunit isoform is included into the calcium channel. Antisense experiments have shown that the same α_1E_ gene encodes for native SNX-482-sensitive and -resistant R-type calcium channels [11]. Six variants of the α_1E_ subunit have been identified and these isoforms present distinct tissue distribution patterns in the central nervous system. For example, SNX-482 acts as a neuroendocrine modulator, inhibiting oxytocin release in vertebrate neurohypophysis and, thus, stopping premature labor during pregnancy. Additional *in vivo* tests have shown the peptide’s antiseizure activity in several models of epilepsy. On the other hand, it does not seem to have any effect on cerebellar granule cells. 

More recent studies not only confirm the specificity and selectivity of SNX-482 for R-type calcium channel blocking but also show that it is rapid and fully irreversible [56]. Furthermore, it appears that SNX-482 also selectively blocks P/Q-type calcium channels at submicromolar concentrations and partially sodium channels.

##### 4.5.2.5. Protoxins I and II

Two peptides, ProTx-I and ProTx-II, from the venom of the green velvet tarantula *Thrixopelma pruniens*, have been isolated and characterized [57]. ProTx-I contain 35 amino acids and ProTx-II contains 30 (Table 8). Both toxins have common structural features including an inhibitory cystine knot motif. ProTx-I and II interact with voltage-gated ion channels (Na_V_ and Ca_V_) and inhibit peak current by shifting the channel voltage dependence of activation to a more depolarized potential [58]. ProTx-II acts in a different way than currently known Na_V_ channel gating modifier toxins. It seems that the binding takes place while the channel is in a closed state. The channel receptor site remains unidentified. ProTx-I and II also potently inhibit some types of voltage gated calcium channels, more precisely, T- and L-type [59]. IC_50_s of 50 nM and 150 nM were recorded for ProTx-I and II, respectively. To summarize, it seems that the toxin-channel interaction surface should be similar in these sodium and calcium channel subtypes.

**Table 8:** Sequences of protoxins I and II [57].

Protoxins	Sequences
ProTx-I	E**C**RYWLGG**C**SAGQT**CC**KHLV**C**SRRHGW**C**VWDGTFS
ProTx-II	Y**C**QKWMWT**C**DSERK**CC**EGMV**C**RLW**C**KKKLW

##### 4.5.2.6. ω-Grammotoxin SIA

Another neurotoxin extracted from spider venom is ω-grammotoxin SIA. This 36 amino acid peptide, found in tarantula *Grammostola spatulata *venom, is a potent blocker of N- and P/Q-type voltage-gated calcium channels [60]. Compared to ω-agatoxin IVA, ω-grammotoxin SIA binds to a distinct part of the P/Q-type calcium channel, thus their effect appears to be additive [61]. Moreover, it appears that the toxin does not block the channel pore but affects the voltage-dependence of activation [62]. 

##### 4.5.2.7. ω-Agatoxins

The venom of the spider *Agelenopsis aperta *was the first source of calcium channel-active spider toxins such as ω-agatoxins. These neuropeptide toxins are presynaptic antagonists of voltage-gated calcium channels. They can be classified according to their structural similarities and their pharmacological properties toward the different types of calcium channels. Type I, II and IV ω-agatoxins block L, N and P/Q currents, respectively, while other ω-agatoxins, like type III, block more than one type of channel. In this review, we will detail this class of neurotoxins, because type IV ω-agatoxins are specific to P/Q-type voltage-gated calcium channels. Type IV ω-agatoxins are important pharmacological tools for understanding P/Q-type calcium channel properties and their role in neurotransmitter release.

## 5. A Special Focus on ω-Agatoxins

American funnel web spiders Agelenopsis aperta, which belong to family of Agelenidae, have gained great interest in neuropharmacology, due to the properties of their venom. The biological activity of spider venoms towards insects was a very first motivation for investigation of spider toxins. Studies which were performed in order to find new insecticides [63] revealed that the substances present in the venom of Agelenopsis aperta alter the properties of voltage-sensitive calcium channels [64]. The venom of Agelenopsis aperta contains several fractions classified in three groups: α-agatoxins, which provoke a postsynaptic block of a transmitter-activated receptor channel, µ-agatoxins that are presynaptic modulators of insect sodium channels [65], and ω-agatoxins, which are presynaptic antagonists of voltage-gated calcium channels. ω-Agatoxins have diverse specificity against various subtypes of calcium channels and show selectivity for calcium channels of various zoological groups (mammalian and insects) [66,67].

### 5.1. Type I and II ω-Agatoxins

ω-Agatoxins of type I are heterodimeric proteins, derived from a single polypeptide chain precursor with five disulfide bonds. Post-translational removal of a heptapeptide leads to the mature form, consisting of two peptide chains: a main 66 amino acid chain possessing four internal disulfide bonds connected via an interchain disulfide linkage to a tripeptide (Table 9). The mature form of ω-agatoxin I has an apparent molecular weight of 7.5 kDa and possesses five tryptophan residues. Up to date, three isoforms of type I ω-agatoxins were identified: ω-agatoxin IA, ω-agatoxin IB and ω-agatoxin IC; all of them have a similar size and share 77% similarity in amino acid sequence with conserved localization of cysteine residues. Type I ω-agatoxins are potent blockers of insect presynaptic calcium channels [66]. Type II ω-agatoxins target different binding sites than type I ω-agatoxins, but on the same type of calcium channels [68]. When type I and II ω-agatoxins are applied together they trigger additive effects and abolish all transmitter release [66]. Structurally, type II ω-agatoxins seems to be large monomeric proteins consisting of 92 amino acid residues, but their full sequence remains unpublished to date (Table 9). Structural features discovered to date, disclose a 43% sequence similarity with the ω-agatoxins I family and identical location of some cysteine residues [64].

**Table 9:** Sequences of ω-agatoxines I and II [64].

ω-Agatoxins	Sequences	Swiss-Prot
IA	AKALPPGSV**C**DGNESD**C**K**C**YGKWHK**C**R**C**PWKWHFTGEGP**C**T**C**EKGMKHT**C**ITKLH**C**PNKAEWGLDW SP**C**	P15969
IB	ERGLPEGAE**C**DGNESD**C**K**C**AGAWIK**C**R**C**PPMWHING	P15970
IIB	G**C**IEIGGD**C**DGYQEKSY**C**Q**CC**RNNGF**C**S	P15971

### 5.2. Type III ω-Agatoxins

Subsequent studies on *Agelenopsis aperta* venom allowed identification of an additional type of ω-agatoxins [27]. Type III ω-agatoxins are 76 amino acid peptides (Table 10), containing 12 cysteine residues forming six internal disulfide bonds, and have an amidated C-terminus [69]. At least seven isoforms of ω-agatoxin IIIA were discovered, having similar amino acid sequences and potencies for channel inhibition. ω-Agatoxin IIIA isoforms are the biggest class of ω-agatoxins with an overall mass around 8.5 kDa [70]. 

**Table 10:** Sequences of ω-agatoxines III [69,70].

ω-Agatoxins	Sequences	Swiss-Prot
IIIA	S**C**IDIGGD**C**DGEKDD**C**Q**CC**RRNGY**C**S**C**YSLFGYLKSG**C**K**C**VVGTSAEFQGI**C**RRKARQ**C**YNSDPDK**C**ESHNKPKRR	P33034
IIIB	S**C**IDFGGD**C**DGEKDD**C**Q**CC**RSNGY**C**S**C**YNLFGYLKSG**C**K**C**EVGTSAEFRRI**C**RRKAKQ**C**YNSDPDK**C**VSVYKPKRR	P81744
IIIC	N**C**IDFGGD**C**DGEKDD**C**Q**CC**XRNGY**C**S**C**YNLFGYLKRG**C**KXEVG	P81745
IIID	S**C**IKIGED**C**DGDKDD**C**Q**CC**RTNGY**C**SXYXLFGYLKSG	P81746

It was found that type III  ω-agatoxins are not very specific peptides. They inhibit all known neuronal HVA calcium currents L-, N-, P/Q- and R-type, but with different affinities. This is probably due to a common binding site on each channel [71]. Type III  ω-agatoxins inhibit P/Q-type calcium currents by up to 40% and are the only peptide ligands that completely block cardiac L-type calcium channels with high affinity [72]. Still, type III ω-agatoxins do not bind to T-type voltage gated calcium channels and, thus, are used for the isolation of pure fractions of these channels. 

A binding site location and a mechanism of current blocking were proposed for type III ω-agatoxins [71]. Taking into consideration that ω-agatoxin IIIA inhibit, in a competitive manner, the binding of ω-conotoxin MVIIC to the N-type calcium channels, and knowing that the binding site of ω-conotoxin is located on the extracellular side of the channel, by analogy, the same position for interaction of ω-agatoxin IIIA was suggested. The simplest possibility of the blocking mechanism is that ω-agatoxin IIIA acts as a leaky lid near the outside of the pore, which reduces calcium current. ω-Agatoxin IIIA is a much larger peptide than ω-conotoxin MVIIC, thus probably fits less tightly within the pore vestibule. This way of action explains why ω-agatoxins IIIA produce incomplete blocking of calcium ion flows while completely eliminating access of ω-conotoxin MVIIC. Taken together, results suggest that ω-agatoxins IIIA inhibit calcium current by blocking the channel pore, whether by direct occlusion or through binding sites that are connected allosterically [61].

### 5.3. Type IV ω-Agatoxins

With the aim of obtaining a mammalian calcium channel antagonist, fractions isolated from the venom of *Agelenopsis aperta* were tested on chicken and rat synaptosomes; this approach resulted in the discovery of type IV ω-agatoxins [73]. Amino acid sequences of IV ω-agatoxins are different from those of types I, II and III ω-agatoxins. Up to now, only three type IV ω-agatoxins have been identified: ω-agatoxins IVA, IVB (also called ω-agatoxin TK) and IVC; however, these are the most important of all four types of ω-agatoxins regarding pharmacological research on P/Q-type calcium channels and on regulation of glutamate release [74]. The major advantage of ω-agatoxins IV is their availability for neurophysiology studies; this is the only type of ω-agatoxin successfully synthesized in a biologically active form [75,76].

#### 5.3.1. Structure of Type IV ω-Agatoxins

All type IV ω-agatoxins are 48 amino acid peptides containing eight cysteines forming four disulfide bridges. Sequence homology between ω-agatoxins IVA and IVB reaches 71% and is represented in Table 11. As for ω-agatoxin IVC, its amino acid sequence is nearly identical with that of ω-agatoxin IVB, possessing the only conformational difference on serine 46, which is L-form, comparing to D-serine in ω-agatoxin IVB.

**Table 11:** Sequences of ω-agatoxins IVA, IVB (containing the D-serine) and IVC.

ω-Agatoxins	Sequence	Swiss-Prot
IVA	KKK**C**IAKDYGR**C**KWGGTP**CC**RGRG**C**I**C**SIMGTN**C**E**C**KPRLIMEGLGLA	P30288
IVB	EDN**C**IAEDYGK**C**TWGGTK**CC**RGRP**C**R**C**SMIGTN**C**E**C**TPRLIMEGLsFA	P37045
IVC	EDN**C**IAEDYGK**C**TWGGTK**CC**RGRP**C**R**C**SMIGTN**C**E**C**TPRLIMEGLSFA	Not available

**Figure 5:**
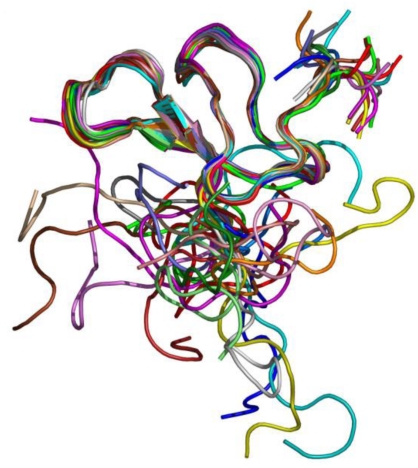
NMR structure of ω-agatoxin IVB [77,78,81].

Experiments of two-dimensional ¹H NMR spectroscopy uncovered the spatial arrangement of ω-agatoxins (Figure 5). Due to its high abundance in spider venom, thus sample accessibility, ω-agatoxin IVB was the first peptide for which a low resolution NMR structure was obtained [77]. Amino acid sequence similarity between ω-agatoxin IVA and IVB suggests possible common structural features and, indeed, disulfide bonding patterns of both molecules are identical [78].

Combination of NMR results with molecular modeling calculations confirm that the molecular scaffold of ω-agatoxins is highly stabilized by the disulfide bridges of identical configuration for both toxins [79]. Additionally, the components of secondary structure of ω-agatoxin IVA were characterized; residues form a short triple-stranded antiparallel β-sheet and three β-turns. The first β-turn is present in the loop between β-strand and N-terminal fragment. The second and third β-turns are located in the long external loop between the first and second β-strand [80].

The disulfide bond between the cysteines 12 and 25 interconnects the first and the second β-strands, while the disulfide bonds between cysteines 4-20 and 19-36 connect the external long loop with the N-terminal region and the third β-strand, respectively. The disulfide bond between cysteines 27 and 34 connects the second and third β-strand [78].

ω-Agatoxin IVA contains only two aromatic residues, tyrosine and tryptophan in positions 9 and 14, respectively, while ω-agatoxin IVB possess three aromatic residues, two of which are homologous to ω-agatoxin IVA and an additional phenylalanine at position 47, located in the C-terminal region. The side chain of tryptophan 14 is located on the upper side of the second β-strand. It should be noted that tyrosine and tryptophan are amphiphilic amino acids that can interact with both hydrophilic and hydrophobic environments [81]. Residues isoleucine in position 5, glycine in 10 and cysteines in position 12, 19, 25, 34 and 36 are located in the peptide core, which is inaccessible for solvents and proteases.

In summary, ω-agatoxins IVA and IVB possess identical disulfide patterns, forming four loop regions, a small three-stranded antiparallel β-sheet, a solvent inaccessible hydrophobic core and disordered N- and C-terminal segments.

#### 5.3.2. Important ω-Agatoxins Residues for Channel Binding

Experiments performed with ω-agatoxins led to several conclusions concerning the characteristic amino acids or whole segments of toxin that are involved in interactions with P-type calcium channels. As a result, different models of toxin binding were proposed. Determination of amino acid sequence and NMR/molecular modeling studies revealed that the basic amino acids residues (arginines in positions 21, 23, 39) form a positively charged surface located close to the hydrophobic C-terminus tail and are conserved in both ω-agatoxins IVA and IVB. Consequently, it was suggested that the positively charged toxin core interacts with calcium channel’s negatively charged residues located near to the external pore [77]. Complementary electrostatic attractions between the toxin and the extracellular loops near the entrance of the pore may contribute to binding affinity, while the toxin’s hydrophobic tail enhances the blockage of the channel by establishing additional molecular contacts with the channel protein [80].

The remarkable difference between ω-agatoxins IVA and IVB is the sequence of amino acids at the N-terminus, which alters charge distribution in the whole molecule; in fact, the two peptides have opposite charges. Whereas the N-terminus of ω-agatoxin IVA is very basic, in ω-agatoxin IVB it is strongly acidic. Electrophysiological analysis of P-type channel inhibition by these two toxins shows that their affinities are similar (2-4 mM). However, kinetics of channel inhibition and dissociation are significantly slower for ω-agatoxin IVB. Experiments indicate that the N-terminus peptide is important in determining the rates at which the toxin binds to and unbinds from the channel. The model explaining the obtained result suggests that the N-terminus sequence of ω-agatoxins may serve to enhance the toxin approach to the outer vestibule of the calcium channel prior to receptor binding. Nevertheless, inhibition potency of the toxins remains unaffected [78].

As biological examinations of a tryptophan 14 modified analogue revealed incomplete channel blocking, it was proposed that this residue might be important for toxin binding to the channel [75]. Tryptophan’s side chain is positioned on the upper side of the second β-strand, thus well exposed for toxin-channel interactions [81]. Currently, there is no defined mechanism explaining how ω-agatoxin IV docks to the channel protein. One cannot help noticing that the model of channel inhibition includes tryptophan 14 as a residue involved in direct binding interactions. In a recent paper presented by our research team, on various synthesized analogues, it was proved that the tryptophan in position 14 is indeed the key-residue for the toxin-channel binding [82].

#### 5.3.3. Importance of Charge for Toxin Interaction

Amino acid sequence examinations revealed that ω-agatoxin IVA is a highly basic molecule, with 10 positively and three negatively charged residues, whereas ω-agatoxin IVB, with six positively charged and six negatively charged residues, is neutral. Taking into consideration that the C- and N-termini are not modified and their participation in the molecule charge is null, the ω-agatoxin IVA net charge is +7 and ω-agatoxin IVB possesses a net charge equal to zero. With such a large difference in the overall charge of the peptides, it could be expected that ω-agatoxin IVB might interact with calcium channels somewhat differently than ω-agatoxin IVA; moreover, the unbinding of ω-agatoxin IVA is strongly voltage-dependent, which might confirm the charge-binding relation. In fact, the properties of calcium channel inhibition by the two toxins are remarkably similar; both are highly selective for the P-type calcium channels; however, their kinetic rate of inhibition is different [77].

#### 5.3.4. Effect of Serine 46 Isomerization in ω-Agatoxin IVB

The post-translational conversion of L- to D-serine described for ω-agatoxin IVB is an evolutionary strategy of stability and activity improvement. Existence of D-amino acids alters the structure of a toxin and is a result of modification of ω-agatoxin IVC via an isomerase [83]. The isomerase activity protects the ω-agatoxin IVB’s C-terminus from degradation; enhanced stability may explain why ω-agatoxin IVB is about 10-times more abundant in venom than the other toxins of its class. A study on both D and L isomers of ω-agatoxins IVB and IVC demonstrated the crucial impact of isomerization on toxin selectivity and stabilization against proteolysis [84,85]. The inhibitory effect comparison of two synthetic ω-agatoxins, one with D-serine: ω-agatoxin IVB, and the second with L-serine: ω-agatoxin IVC, revealed that the L-isomer is 90-fold less potent [86].

#### 5.3.5. Action Mode and Location of the Binding Site

ω-Agatoxins IV are the most potent blockers of P-type calcium channels in both insect and mammalian central neurons. However, the ω-agatoxins IVA and IVB constitute a unique component of *Agelenopsis aperta* venom that has remarkable specificity for P-type calcium channels in the mammalian central nervous system. These channels occur throughout the brain, but are particularly developed in Purkinje cells of the cerebellum and are directly involved in glutamate release.

The mechanism of toxin-channel interaction of ω-agatoxin IVA on P/Q-type calcium channels is voltage-dependent, meaning that the toxin perturbs the gating properties of the channel and does not physically occlude the pore. Binding of the toxin shifts the channel’s activation voltage to positive potentials, which are not reached during normal physiological activity of the cell. The toxin raises the energy barrier for voltage-dependent gating to non-physiological levels. Type IV ω-agatoxins bind with high affinity to closed state channels, leaving the channels in the open state unaffected [87]. The experiments showed that repetitive application of strongly positive voltage steps led to the opening of the channel and the dissociation of the toxin [88]. Toxin dissociation occurs because, in contrast to its high affinity binding to the channel in the closed state, the affinity of the channel in its open state is very low. Additionally, strong depolarization may cause channel conformational changes, which are unfavorable from the point of view of spatial arrangement and act allosterically on the toxin binding site [87]. Thus, ω-agatoxin IVA inhibits the inward calcium current by increasing the voltage required for channel activation, without affecting the permeation of the channel.

In order to establish the binding site’s location on the calcium channel, comparative experiments using ω-agatoxin IVA and ω-grammotoxin SIA were performed. A similar shift of gating on the N- and P-type calcium channels was observed, suggesting similar binding sites for both toxins. The results of electrophysiological studies show that the binding sites for both toxins involve a linker between transmembrane segments 3 and 4 in P-type calcium channels. However, the voltage shifts produced by the two toxins is additive, suggesting that they bind simultaneously to different sites of the channel [61].

Sequence comparison between the α_1_ subunit of voltage-gated potassium, sodium and calcium channels performed in order to establish the similarities between the regions of toxin binding, allowed the amino acids that are probably involved in channel-toxin interactions to be chosen. Subsequent mutation of the channel protein revealed, at least partially, the location of the binding site for ω-agatoxin IVA, which is situated on the external part of the channel near the channel pore. The extracellular loop between transmembrane segments 3 and 4 in domain IV of the channel is directly affected by ω-agatoxin IVA. This places the toxin in direct proximity to the transmembrane segment 4 voltage sensor. Replacement of glutamic acid with lysine, at position 1658 in the channel protein, makes the channel completely resistant to modifications provoked by ω-agatoxin IVA [89]. These results provide compelling insight into the manner by which gating modification triggered by toxins blocks calcium channels.

#### 5.3.6. Selectivity and Affinity

Selectivity of ω-agatoxin IVA for the P-type calcium channels, which are responsible for the release of glutamate, is well established, with inhibition occurring at concentrations less than 10 nM and a K_d_ of 2 nM. ω-Agatoxin IVB evokes the same selectivity and similar potency with a K_d_ of 3 nM, nevertheless, is about eight-times slower regarding the kinetics of current blocking. Given that the kinetics of inhibition is dependent on toxin concentration, the ten-fold higher abundance of ω-agatoxin IVB in venom equilibrates the rate of block, which is comparable to that of ω-agatoxin IVA. As previously mentioned, biological activity of ω-agatoxin IVC, precursor of ω-agatoxin IVB, is much less important than that of ω-agatoxin IVB.

Regarding Q-type calcium channels, the selectivity of ω-agatoxins is less remarkable (K_d_ ~100 nM), with an effective toxin concentration arising to a micromolar range. Studies of the effect of ω-agatoxin IVA on the N-type calcium currents demonstrated that the selectivity towards P/Q-type currents is not complete. Subsequent investigation showed that ω-agatoxin IVA affects a variety of dihydropyridine-insensitive calcium channels (non L-type) when used at micromolar range. These data suggest that ω-agatoxin IVA is selective at the nanomolar range; nevertheless, its selectivity is diminished at micromolar range, thus its usefulness for functional studies of Q-type calcium channels is limited [90]. The dissimilarity of selectivity towards P- and Q-type channels is explained by the different structures of the channels, resulting from differential splicing of the genes encoding for the α_1A_ subunit [67].

Notwithstanding this limitation, the ω-agatoxins IVA and IVB retain their usefulness as selective and potent blockers of neuronal P-type voltage-gated calcium channels and will remain important tools to relate the properties of recombinant calcium channels to the complexity of their native counterparts.

Since the tryptophan residue located at position 14 of the ω-agatoxin IVB has been suggested as essential for binding, analogues were synthesized. Our recent studies of constraint analogues of the loop containing the tryptophan 14 revealed a potent agonist activity on P/Q-type calcium channels [82]. The synthesis of such cyclopeptides is currently under development. Furthermore, specific modifications increasing the peptide stability *in vitro* and *in vivo* are being explored.

## 6. Conclusion

In this review, a large variety of natural potential tools were presented; the described peptide neurotoxins and their analogues helped in the elucidation of structure/function relationships toward voltage-gated calcium channels. Furthermore, the use of synthetic analogues of these toxins will lead to the development of new therapeutic agents and strategies for treatment of ion channel-related diseases.
